# First-Principles Investigation on Ru-Doped Janus WSSe Monolayer for Adsorption of Dissolved Gases in Transformer Oil: A Novel Sensing Candidate Exploration

**DOI:** 10.3390/s24185967

**Published:** 2024-09-14

**Authors:** Liang Cao, Ruilong Ma, Mingxin Ran, Hao Cui

**Affiliations:** 1 College of Engineering and Technology, Southwest University, Chongqing 400715, China; liangcao2020@swu.edu.cn; 2 College of Artificial Intelligence, Southwest University, Chongqing 400715, China; maruilong@email.swu.edu.cn (R.M.); rmx112603@email.swu.edu.cn (M.R.)

**Keywords:** Ru-WSSe monolayer, oil-immersed transformers, first-principles theory, gas sensor

## Abstract

Using first-principles theory, this work purposes Ru-doped Janus WSSe (Ru-WSSe) monolayer as a potential gas sensor for detection of three typical gas species (CO, C_2_H_2_, and C_2_H_4_), in order to evaluate the operation status of the oil-immersed transformers. The Ru-doping behavior on the WSSe surface is analyzed, giving rise to the preferred doping site by the replacement of a Se atom with the formation energy of 0.01 eV. The gas adsorption of three gas species onto the Ru-WSSe monolayer is conducted, and chemisorption is identified for all three gas systems with the adsorption energy following the order: CO (−2.22 eV) > C_2_H_2_ (−2.01 eV) > C_2_H_4_ (−1.70 eV). Also, the modulated electronic properties and the frontier molecular orbital are investigated to uncover the sensing mechanism of Ru-WSSe monolayer upon three typical gases. Results reveal that the sensing responses of the Ru-WSSe monolayer, based on the variation of energy gap, to CO, C_2_H_2_, and C_2_H_4_ molecules are calculated to be 1.67 × 10^6^, 2.10 × 10^5^, and 9.61 × 10^3^, respectively. Finally, the impact of the existence of O_2_ molecule for gas adsorption and sensing is also analyzed to uncover the potential of Ru-WSSe monolayer for practical application in the air atmosphere. The obtained high electrical responses manifest strong potential as a resistive sensor for detection of three gases. The findings hold practical implications for the development of novel gas sensing materials based on Janus WSSe monolayer. We anticipate that our results will inspire further research in this domain, particularly for applications in electrical engineering where the reliable detection of fault gases is paramount for maintaining the integrity and safety of power systems.

## 1. Introduction

Oil-immersed transformers, being the backbone of electrical grids, play a significant role in completing the electricity conversion and transmission for the power system [1]. As key components of the electrical grid, oil-immersed transformers require meticulous monitoring to maintain their integrity and ensure uninterrupted power supply. Over time, various factors such as electrical stress, thermal degradation and mechanical faults can lead to the decomposition of the transformer oil, followed by the generation of gases within the transformer oil [2]. In this regard, the safe and stable operation status of such electrical equipment could be weakened [3,4], thus threatening the safe operation of the power system accordingly [5,6]. These typical gases, primarily CO, C_2_H_2_, and C_2_H_4_, can provide valuable insights into the transformer’s condition [7,8]. By analyzing the concentration and ratios of these gases, experts can detect potential issues such as overheating, arcing, or other internal faults that might lead to equipment failure [9,10]. This technique is so-called dissolved gas analysis (DGA), which provides a non-intrusive method to evaluate the internal condition of transformers by measuring the concentration and composition of gases dissolved in the oil [11]. Besides, this technique not only helps in predictive maintenance but also extends the lifespan of the transformers, ensuring uninterrupted power supply and reducing unexpected outages. 

With the progress of nanomaterials and related gas sensing techniques, nanomaterial-based gas sensors for DGA are purposed and have been deemed a novel and workable manner with the advantages of high sensitivity, rapid response, and low cost [12,13]. Nowadays, transition-metal dichalcogenides (TMDs) as a novel group of 2D materials have garnered significant attention in the fields of gas adsorption and sensing [14,15,16], due to their advantageous qualities such as a large surface area, high electron mobility, and tunable semiconducting properties [17,18,19]. Recently, Janus TMDCs have recently emerged as a promising class of gas-sensitive sensing materials. These unique materials have an asymmetric structure where a transition metal layer is sandwiched between two different chalcogen layers. The large surface area, combined with the high chemical reactivity stemming from the asymmetric structure, allows Janus TMDs to efficiently adsorb and interact with gas molecules [20]. This interaction leads to changes in the material’s electronic properties, which can be precisely measured and correlated to the presence and concentration of specific gases. The most popular Janus TMDs is MoSSe, which has been theoretically proven with excellent sensing capability upon gas molecules [21,22], and the monolayer of MoSSe decorated by metal atoms behaves admirable adsorption and sensing performances upon CO and NO [23]. Apart from that, theoretical studies reveal that a WSSe monolayer decorated by metal atoms exhibits exceptional CO sensing properties [24]. One should recognize that metal-doped WSSe monolayer as a typical gas sensor is less discovered, and theoretical investigation on this issue could significantly stimulate the capability of WSSe-based nanomaterials, thus paving the way for further and deeper research upon Janus TMDs. 

In this report, first-principles theory is employed to conduct the adsorption process of Ru-doped WSSe (Ru-WSSe) monolayer on CO, C_2_H_2_, and C_2_H_4_ molecules. The ruthenium (Ru) atom is selected as the metal dopant in this work because of its good catalytic activity in the nanosystems with the gas species, which has been verified in the previous references [25,26]. The gas adsorption process of three typical dissolved gas species onto the Ru-WSSe monolayer can not only highlight the adsorption characteristic or gas-surface interaction between them, but also illustrate the deformed electronic property of the Ru-doping in such interaction from the atomic and molecular aspects. These analyses would be beneficial in uncovering the gas sensing mechanism and assessing the potential gas sensing properties for this novel Janus material [27]. The findings in this work can not only contribute to the advancement of 2D Janus TMDs for gas sensing applications, but also hold significant implications for equipment defect evaluation in the power system.

## 2. Computational Details

In the current investigation, the execution of all first-principles simulations was facilitated by the DMol^3^ computational package [28], which was nestled within the generalized gradient approximation (GGA) framework and instrumental in deploying the Perdew–Burke–Ernzerhof (PBE) functional to meticulously manage the intricacies of electron exchange and correlation [29,30]. To address the subtleties of Van der Waals forces and their long-range interactions, Grimme’s DFT-D3 method was seamlessly integrated, adopting an unrestricted spin-polarized methodology [31]. Further augmenting our computational strategy, the double numerical plus (DNP) polarization was meticulously chosen as the foundational basis set for atomic orbitals, while the DFT semi-core pseudopotential (DSSP) method was adeptly applied to mitigate the relativistic effects inherent in metal atoms [32]. In the quest for precision in geometric optimization and electronic structure calculations, a k-point mesh of 10 × 10 × 1 was meticulously selected [33]. With an unwavering commitment to the fidelity of total energy calculations, a self-consistent field energy convergence threshold of 10^−6^ Ha was established, alongside a global orbital cutoff radius of 5.0 Å and a judicious smearing value of 0.005 Ha [34]. Moreover, the convergence criterion for geometric optimizations was set with a stringent standard of 10^−4^ Ha [35].

The construction of a 3 × 3 × 1 WSSe supercell, encompassing a total of 27 atoms—9 W, 9 S, and 9 Se atoms—was meticulously crafted to function as a nano-substrate for the doping of the Ru atom and the subsequent processes of gas adsorption [36]. To preemptively diminish any potential interface interactions, a vacuum region spanning 20 Å was strategically incorporated along the *z*-axis of the WSSe superlattice. Additionally, insightful Hirshfeld analysis was engaged to determine the atomic charge of the Ru dopant (*Q*_Ru_) within the Ru-WSSe system and the molecular charge (*Q*_T_) of the absorbed gas molecules. The positivity or negativity of *Q*_Ru_ or *Q*_T_, respectively, delineates the electron-donating or electron-accepting characteristics of the system.

## 3. Results and Discussion

### 3.1. Analysis of Ru-WSSe Monolayer

In this work, the Ru-WSSe monolayer is established by replacing an S or Se atom within the WSSe surface with a Ru atom, after which the formation energy (*E*_form_) is calculated to identify the preferred Ru-doping configuration on the pristine Janus WSSe monolayer. It should be noted that the *E*_form_ is calculated to determine the required energy for Ru-doping on the WSSe monolayer, expressed as [35]:

(1) $$E_{\mathrm{form}}=E_{\text{Ru-WSSe}}-E_{\mathrm{WSSe}}-\mu_{\mathrm{Ru}}+\mu_{\text{S/Se}}$$

in which $E_{\text{Ru-WSSe}}$ and $E_{\mathrm{WSSe}}$ are the energies of Ru-doped and intrinsic WSSe monolayer, respectively, whereas *μ*_Ru_ and *μ*_S/Se_ are the chemical potential of the Ru dopant and the S/Se atom. 

**Figure 1:**
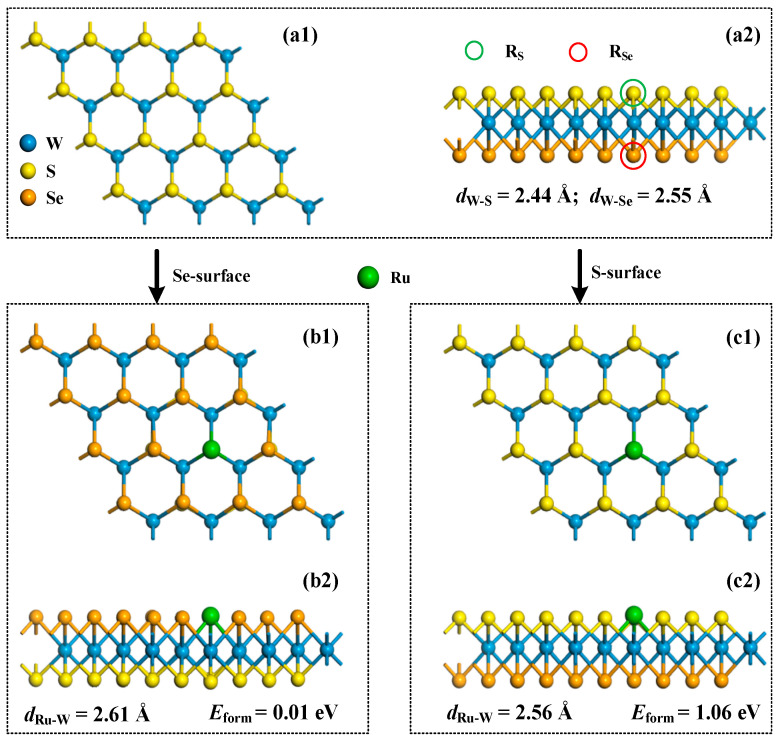
Configurations of pristine Janus WSSe monolayer (**a1**,**a2**), and Ru-doping from the Se-surface (**b1**,**b2**) and from the S-surface (**c1**,**c2**) of the WSSe monolayer.

To compare the geometric structures of pristine and Ru-doped WSSe monolayer (via S and Se atomic layer), we exhibit their configurations in Figure 1 to understand the geometric deformation of WSSe monolayer caused by Ru-doping. Firstly, focusing on the morphology of pristine WSSe monolayer in Figure 1(a1,a2), it is evident that the Se atomic layer is somewhat more distant from the W atomic layer than the S atomic layer. Indeed, the W-Se bond length measures 2.55 Å, which is marginally longer than the W-S bond length of 2.44 Å. This difference can be attributed to the larger atomic radius of Se compared to S [37], resulting in the increased layer distance from the W atomic layer. Vibrational analysis indicates that the frequency range for the pristine WSSe monolayer spans from 273.21 to 526.95 cm^−1^. The absence of any imaginary frequencies in this range confirms the excellent chemical stability of the pristine WSSe monolayer. Additionally, the lattice constant of the pristine Janus WSSe monolayer has been calculated to be 3.26 Å, aligning well with the previously reported value of 3.25 Å from studies [38,39]. 

Then we emphasize the Ru-doping behavior on the Janus WSSe monolayer. As illustrated in Figure 1(b1,b2), Ru-doping on the Se-surface results in the Ru atom slightly protruding from the Se layer, with a measured Ru-W bond length of 2.61 Å. This is a 0.06 Å elongation compared to the W-Se bond. Similarly, Figure 1(c1,c2) shows Ru doping on the S-surface, where the Ru dopant also protrudes, with a Ru-W bond length of 2.56 Å, which is 0.12 Å longer than the W-S bond. Notably, the Ru-W bond in both configurations is significantly shorter than the sum of the covalent radii of Ru and W (2.62 Å [37]), indicating a strong binding force between the Ru and W atoms, characteristic of covalent bonding [40]. Also, it should be mentioned that the morphology of the WSSe monolayer suffers little deformation after Ru-doping, wherein the W-Se and W-S bonds are measured to be 2.55 and 2.44 Å, respectively, which are equivalent to the pristine counterpart. Furthermore, the *E*_form_ for Ru-doping on the Se- and S-surfaces is calculated to be 0.01 and 1.06 eV, respectively. This suggests that doping with Ru on the Se-surface requires less energy, implying that the Ru dopant is more readily incorporated into the WSSe monolayer by replacing a Se atom, exhibiting a stronger binding force with the W atoms compared to the replacement of an S atom [41]. Besides, one can infer that the Ru-doping by the replacement of a Se atom is indicative of a highly favorable and energetically stable doping process. Such minimal energy requirement for this substitution suggests that the Ru dopant can be readily incorporated into the WSSe lattice without significantly disrupting its structural integrity. Based on these findings, we select the configuration depicted in Figure 1(b1,b2) as the definitive Ru-WSSe monolayer for subsequent analysis of gas adsorption and sensing capabilities. To further confirm the chemical stability of the Ru-WSSe monolayer, vibrational analysis was conducted. The analysis revealed a frequency range from 73.79 to 834.21 cm^−1^, with no imaginary frequencies, which supports the conclusion of its good chemical stability [42].

**Figure 2:**
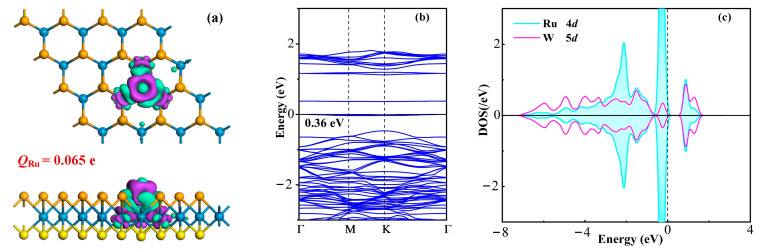
(**a**) CDD of Ru-WSSe monolayer, (**b**) BS of Ru-WSSe monolayer, and (**c**) orbital DOS of Ru and W atoms. In CDD, the cyan and violet areas are electron accumulation and depletion, respectively, with the isosurface of 0.02 e/Å^3^. In BS, the black value is the bandgap of Ru-WSSe monolayer.

To delve into the electron distribution and electronic properties of the Ru-WSSe monolayer, we have plotted the charge density difference (CDD) and band structure (BS) for the Ru-WSSe monolayer. Additionally, the density of states (DOS) for the Ru and W atoms has been charted to provide a deeper understanding of the bonding nature within the Ru-WSSe monolayer. The visual representations in Figure 2 are instrumental in elucidating the altered electronic properties of the Janus WSSe monolayer due to Ru-doping.

As shown in Figure 2a by the CDD, electron depletion is primarily observed around the Ru dopant, while electron accumulation is predominantly found at the Ru-W bonds. This electron distribution in the CDD indicates the electron-donating characteristic of the Ru dopant and the strong binding affinity between the Ru and W atoms. The electron-donating nature of the Ru dopant is further substantiated by Hirshfeld analysis, which indicates a charge transfer of 0.065 e from the Ru dopant to the W atom of the WSSe monolayer. On the contrary, the replaced Se atom in the pristine WSSe monolayer is charged by −0.025 e, indicating the charge-transfer from the W atom to the Se atom. That is, Ru-doping modulates the electron distribution within the WSSe monolayer thus bringing about the deformation in its electronic property [43]. These charge-transfers can be attributed to the larger electronegativity of the Se atom (2.55) compared with the W and Ru atoms (2.36 and 2.28, respectively) [44], making the stronger electron affinity of the Se atom when bonding with W and Ru atoms. Regarding the BS of the Ru-WSSe system, as illustrated in Figure 2b, the bandgap is determined to be 0.36 eV, with both the top valence band and bottom conduction band situated at the K point. In contrast to the pristine Janus WSSe monolayer’s direct semiconducting nature with a bandgap of 1.71 eV as reported in Reference [38], it can be deduced that Ru-doping significantly narrows the bandgap by 1.35 eV, while preserving its direct semiconducting property. Additionally, the strong binding force between the Ru and W atoms is evident from the atomic DOS depicted in Figure 2c. This figure illustrates that the Ru 4*d* orbital is substantially hybridized with the W 5*d* orbital in the energy ranges of −6.9 to 0 and 0.6 to 1.5 eV. Such electron hybridizations confirm the robust orbital interaction between the Ru and W atoms, which is responsible for the formation of covalent Ru-W bonds [45]. 

### 3.2. Gas Adsorptions on Ru-WSSe Monolayer

**Figure 3:**
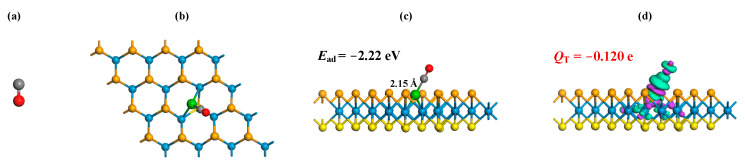
CO molecule (**a**), related gas adsorption configurations (**b**,**c**) and related CDD (**d**). In CDD, the settings are the same as Figure 2.

**Figure 4:**
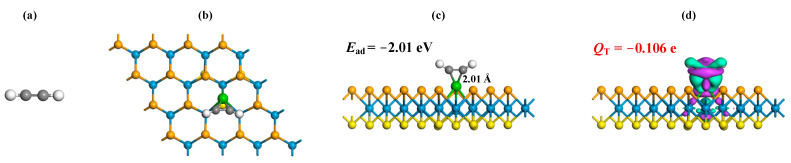
C_2_H_2_ molecule (**a**), related gas adsorption configurations (**b**,**c**) and related CDD (**d**). In CDD, the settings are the same as Figure 2.

**Figure 5:**
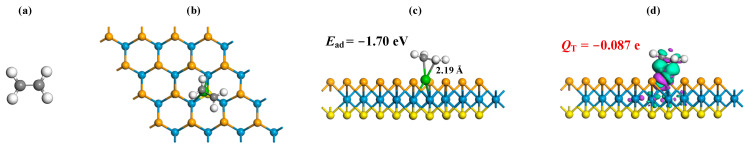
C_2_H_4_ molecule (**a**), related gas adsorption configurations (**b**,**c**) and related CDD (**d**). In CDD, the set are the same as Figure 2.

The gas adsorption processes for three representative gas species are conducted above the Ru atom in the characterized Ru-WSSe monolayer, with the atomic distance (specifically, the initial adsorption distance, denoted as d) set to the appropriate value of 2.5 Å. Additionally, the gas species approach the Ru atom through various configurations to ascertain the most stable configuration (MSC) for their adsorption. Concurrently, the MSC can be identified by calculating the adsorption energy (*E*_ad_), which is the most negative among the different adsorption configurations. This is based on the principle that a more negative *E*_ad_ indicates a stronger adsorption performance in the gas-surface interactions. For the analysis of gas adsorption configurations, we focus solely on the MSC in the main text, where *E*_ad_ is calculated as follows [46]:

(2) $$E_{\mathrm{ad}}=E_{\text{Ru-WSSe/gas}}-E_{\text{Ru-WSSe}}-E_{\mathrm{gas}}$$

where $E_{\text{Ru-WSSe/gas}}$, $E_{\text{Ru-WSSe}}$ and $E_{\mathrm{gas}}$ signify the gas-adsorbed Ru-WSSe system, the pure Ru-WSSe monolayer, and the isolated gas molecule, respectively. Through meticulous geometric optimizations, the MSC for the adsorption of CO, C_2_H_2_ and C_2_H_4_ adsorption onto the Ru-WSSe monolayer are illustrated in Figure 3, Figure 4 and Figure 5, respectively. Additionally, the CDD of the relevant MSC is presented to provide a deeper understanding of the charge-transfer dynamics and the intrinsic nature of the bonding during the interaction between the gas molecules and the Ru-WSSe monolayer. This visual representation of the CDD enhances our comprehension of the electronic interactions at play, offering a nuanced perspective on the adsorption process. 

Firstly, the adsorption of CO molecule onto the Ru-WSSe monolayer, as plotted in Figure 3, is a strong gas-surface interaction that can be visually described as the C atom of the CO molecule being ensnared by the Ru dopant. This results in the formation of a Ru-C bond, characterized by an optimal length of 2.15 Å. Simultaneously, the CO molecule hovers above the Ru dopant, creating an incline with respect to the WSSe surface, suggesting a distinct orientation in the adsorption process. Such configuration allows us to infer that the Ru dopant exerts a stronger binding affinity with the carbon atom of the CO molecule, as opposed to the oxygen atom, leading to the formation of a Ru-C bond rather than a Ru-O bond. The calculated sum of the covalent atomic radii for ruthenium and carbon is 2.22 Å [37], which underscores the robust binding force between the Ru dopant and the carbon atom, indicative of a covalent Ru-C bond. Further analysis of the adsorbed system reveals an *E*_ad_ of −2.22 eV, a value that not only substantiates the strong binding interaction between the Ru-WSSe monolayer and the CO molecule but also confirms the occurrence of chemisorption. This is evidenced by the absolute value of *E*_ad_ exceeding the threshold of 0.8 eV, a critical benchmark for identifying such interactions [47]. Delving deeper into the CDD analysis, we observe electron accumulations specifically localized around the Ru-C bond. This finding provides compelling evidence of a strong binding force, where electron hybridization is taking place, reinforcing the covalent nature of the Ru-C bond. Lastly, the Hirshfeld analysis yields an intriguing insight: the adsorbed CO molecule carries a charge of −0.120 e. This observation points to the electron accepting characteristic of the CO molecule upon adsorption onto the Ru-WSSe monolayer, further enriching our understanding of the electronic interactions in this system. 

Further, the adsorption of C_2_H_2_ molecule on the Ru-WSSe monolayer, as elegantly portrayed in Figure 4, unveils an intriguing scenario where the two carbon atoms of the C_2_H_2_ molecule are both ensnared by the Ru dopant, resulting in the formation of two symmetrical Ru-C bonds, each with an equal length of 2.01 Å. This interaction induces a significant geometric transformation in the adsorbed C_2_H_2_ molecule, where the C-H bonds are displaced upwards into the vacuum region, thus transforming the molecule from a linear to a non-linear configuration. The observed Ru-C bond length is intriguingly shorter than the sum of the covalent atomic radii of Ru and C atoms, a phenomenon that underscores the intense binding force within the C_2_H_2_ adsorbed system that gives rise to the formation of covalent Ru-C bonds [48]. Besides, the *E*_ad_ of −2.01 eV further substantiates the robust chemisorption of the Ru-WSSe monolayer upon the C_2_H_2_ molecule, highlighting the favorable binding force in this gas-surface interaction [47]. The CDD distribution reveals electron accumulations encircling the Ru-C bonds, which is a visual testament to the electron localization and the strong binding force that characterizes their formation. In stark contrast, the C=C double bond of the C_2_H_2_ molecule is surrounded by electron depletion, indicating a weakened binding force post-adsorption. This accounts for the molecule’s deformation and distortion, as it adapts to the surface. Employing the Hirshfeld method, it is determined that the adsorbed C_2_H_2_ molecule carries a charge of −0.106 e. This signifies that during the adsorption process, the Ru-WSSe monolayer transfers 0.106 e to the C_2_H_2_ molecule, thereby highlighting the electron-accepting nature of the C_2_H_2_ molecule in this interaction.

In the case of C_2_H_4_ adsorption onto the Ru-WSSe monolayer, we observe a similar configuration to the C_2_H_2_ system where the two carbon atoms of the C_2_H_4_ molecule are captured by the Ru dopant. However, unlike the planar alignment seen in C_2_H_2_ system, the adsorbed C_2_H_4_ molecule adopts a tilted orientation with respect to the WSSe surface. This results in the formation of two Ru-C bonds with distinct lengths, precisely measured at 2.19 and 2.21 Å, respectively. This variation in bond length is indicative of the asymmetrical interaction at play. Post-adsorption, the C_2_H_4_ molecule undergoes a morphological transformation, deviating from its original planar structure to exhibit a non-planar conformation. This alteration suggests a high degree of interaction with the surface, indicative of favorable adsorption characteristics. The *E*_ad_ for this system is calculated to be −1.70 eV, a value that underscores the chemisorption nature of the C_2_H_4_ molecule’s interaction with the Ru-WSSe monolayer. The Hirshfeld analysis provides further insight, revealing a charge transfer of −0.087 e to the adsorbed C_2_H_4_ molecule. This charge transfer pathway from the Ru-WSSe monolayer to the C_2_H_4_ molecule is a testament to the electron-accepting behavior of the adsorbed molecule. Additionally, the CDD analysis elucidates the electron distribution within the system. There is a notable concentration of electron density along the Ru-C bonds, signifying the strong binding forces at these sites. Conversely, the Ru dopant exhibits electron depletion, suggesting a transfer of charge from the dopant to the C_2_H_4_ molecule. These electron accumulations and depletions are the hallmarks of the covalent character of the Ru-C bonds and the electron transfer process that strengthens the adsorption.

It is worth noting that the adsorption configurations of Ru-WSSe monolayer upon three gas species paint a detailed picture of the complex interplay, emphasizing the geometric changes, electron transfer, and the resultant strong binding forces that define this adsorption process. Also, the comprehensive analysis of the adsorption of three prototypical gas species—CO, C_2_H_2_, and C_2_H_4_—in oil-immersed transformers reveals a hierarchy of chemisorptive behavior, with CO exhibiting the strongest affinity, followed by C_2_H_2_, and then C_2_H_4_. This ordered adsorption performance not only highlights the distinct interactions between each gas molecule and the Ru-WSSe monolayer but also underscores the importance of molecular structure in determining adsorption strength. In all three systems, the charge-transfer pathway is consistently from the Ru-WSSe monolayer to the respective gas species, revealing a common electron-accepting property of the CO, C_2_H_2_, and C_2_H_4_ molecules. This charge transfer is pivotal as it leads to electron re-distributions within the adsorbed systems, effectively modulating the electronic properties of the Ru-WSSe monolayer. Such modulation is crucial for understanding the underlying mechanisms that govern the adsorption processes and could potentially influence the catalytic activity and sensitivity of the monolayer.

### 3.3. Analysis of Electronic Property in Gas Adsorbed Ru-WSSe Systems

**Figure 6:**
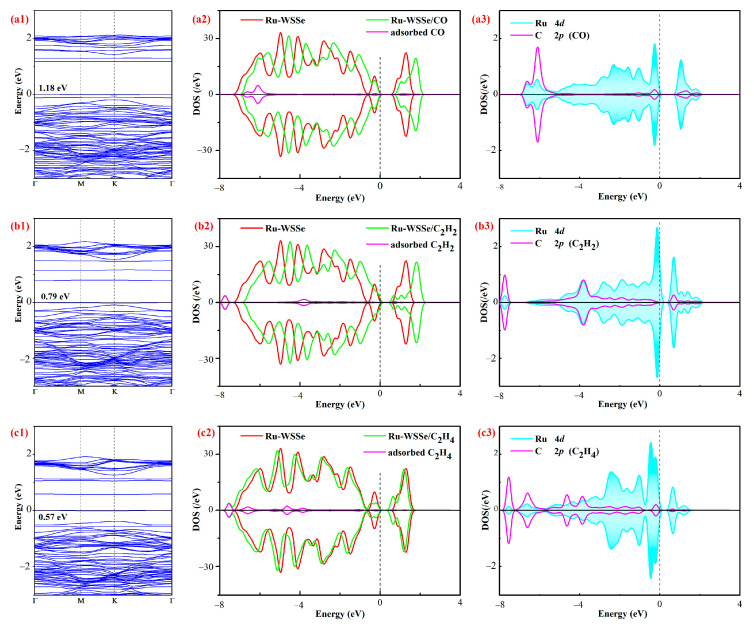
Electronic properties of gas adsorbed Ru-WSSe systems. (**a1**–**a3**) CO system, (**b1**–**b3**) C_2_H_2_ system and (**c1**–**c3**) C_2_H_4_ system. The settings here are the same as Figure 2.

In the realm of gas adsorptions, the phenomenon of charge-transfer plays a pivotal role in influencing the electronic properties of the Ru-WSSe monolayer. In this section, we aim to delve deeper into the intricate electronic modulations induced by the adsorption of these gas species. A detailed examination of these modulated electronic properties is essential for a thorough understanding of the gas adsorption processes and their implications in practical applications for the purposed Ru-WSSe monolayer, such as in the monitoring and maintenance of oil-immersed transformers. Specifically, the BS of the gas adsorbed systems, a comparative analysis of the total DOS for the Ru-WSSe monolayer before and after gas adsorption, and the orbital DOS of the atoms involved in the bonding within the gas-adsorbed systems are exhibited in Figure 6. At first glance, the DOS for spin-up and spin-down in all gas adsorbed systems appear symmetrical, indicating that the Ru-WSSe monolayer retains its non-magnetic property throughout the three gas adsorption processes. In essence, the adsorption of three typical gas of transformer oil does not introduce any magnetic moment to the Ru-WSSe monolayer.

The analysis of the BS distributions for the three gas adsorbed systems reveals the intriguing changes in the bandgap of the Ru-WSSe monolayer upon adsorption of CO, C_2_H_2_, and C_2_H_4_. Specifically, the bandgaps for the monolayer with CO, C_2_H_2_, and C_2_H_4_ adsorbed are calculated to be 1.18 eV, 0.79 eV, and 0.57 eV, respectively. This indicates a significant increase in the bandgap compared to the isolated Ru-WSSe monolayer, which has a bandgap of 0.36 eV. The increments are substantial, amounting to 0.82 eV for CO, 0.43 eV for C_2_H_2_, and 0.21 eV for C_2_H_4_. These findings suggest that the electrical conductivity of the Ru-WSSe monolayer could be substantially reduced following the adsorption of these gas species, with varying degrees of impact [49]. These findings are significant in understanding the electronic structure modifications that occur upon gas adsorption on the Ru-WSSe monolayer, potentially enhancing its utility as a material for gas sensing applications. 

This hypothesis is supported by a comparative analysis of the total DOS for the isolated and gas adsorbed Ru-WSSe monolayers. The total DOS comparison indicates that the electronic states introduced by the adsorbed gas species—CO, C_2_H_2_, and C_2_H_4_—contribute to the overall DOS of the monolayer. It is also important to highlight that the states of the adsorbed gas molecules near the Fermi level have a pronounced effect on the electronic properties of the Ru-WSSe monolayer. Furthermore, the states at the bottom of the conduction band of the Ru-WSSe monolayer are observed to shift to higher energies post-adsorption. This shift is associated with the electron-accepting behavior of the gas molecules during adsorptions, thereby increasing the bandgap for the gas adsorbed systems [50]. These observations are crucial for understanding the electronic behavior of Ru-WSSe monolayer and its potential applications in gas sensing technology.

Concurrently, the orbital DOS for the atoms involved in bonding within the three gas adsorbed systems reveals significant state overlap and orbital hybridization between the Ru dopant and the C atoms of the adsorbed gas species. Notably, the Ru 4*d* orbital exhibits extensive hybridization with the C 2*p* orbitals of the CO molecule at energies of −6.6, −6.1, −1.0, −0.3, 1.0, and 1.7 eV; with the C_2_H_2_ molecule at energies of −7.7, −6.5~−0.1, 0.7, 1.1, and 1.4 eV; and with the C_2_H_4_ molecule at energies of −7.5, −6.8, −5.4~−0.2, and 0.6. These hybridizations underscore the robust orbital interactions between the Ru dopant and the carbon atoms of the three representative gas species, which are likely responsible for the strong binding forces observed in the Ru-C bonds, as indicated by the favorable *E*_ad_ within the gas adsorption systems. Furthermore, it is crucial to recognize the pivotal role of the Ru dopant in the formation of Ru-C bonds. The states contributed by the Ru dopant, particularly those in the vicinity of the Fermi level, are predominant in the electron hybridization process [51]. This observation is instrumental in understanding the importance of the Ru dopant in the whole monolayer, thereby influencing its reactivity towards different gas molecules. 

### 3.4. Resistive Gas Sensing Exploration

Given the pronounced modulation of the electronic properties in the Ru-WSSe monolayer due to the adsorption of three typical gas species commonly found in oil-immersed transformers—CO, C_2_H_2_, and C_2_H_4_—the bandgap of the monolayer has been observed to expand to varying degrees. This alteration in the bandgap is pivotal for the material’s potential application in gas sensing, as it directly influences the electrical conductivity of the sensing material. The modification in bandgap can lead to a corresponding change in the electrical conductivity, which is a critical factor for the sensitivity and selectivity of gas sensors.

Beyond the bandgap, the energy gap is another significant parameter in assessing the electrical conductivity of a material, which is derived from the frontier molecular orbital (FMO) theory [52]. This theory hinges on the identification of the highest occupied molecular orbital (HOMO) and the lowest unoccupied molecular orbital (LUMO), with the energy gap defined as the energy difference between these two critical orbitals. The relationship between electrical conductivity (*σ*) and the energy gap (*E*_g_) is fundamental to understanding how the material will respond to the presence of different gases. This relationship can be mathematically expressed and is often described by the following equation [53]:

(3) $$\sigma =\lambda \cdot e^{\left(-E_{g}/2kT\right)}$$

In Equation (3), *T* is absolute temperature, *k* is the Boltzmann constant, and λ is a constant. The application of the energy gap serves a dual purpose in this work. Firstly, it provides a means to verify the precision of the determined bandgap for the Ru-WSSe monolayer and gas adsorbed systems. Secondly, it enables the calculation of the sensing response for the Ru-WSSe monolayer when utilized as a resistive gas sensor. This is a crucial metric for assessing the performance of Ru-WSSe monolayer in detecting the presence of specific gases, calculated as [54]:

(4) $$S=\left({\sigma_{\mathrm{gas}}}^{-1}-{\sigma_{\mathrm{pure}}}^{-1}\right)/{\sigma_{\mathrm{pure}}}^{-1}$$

**Figure 7:**
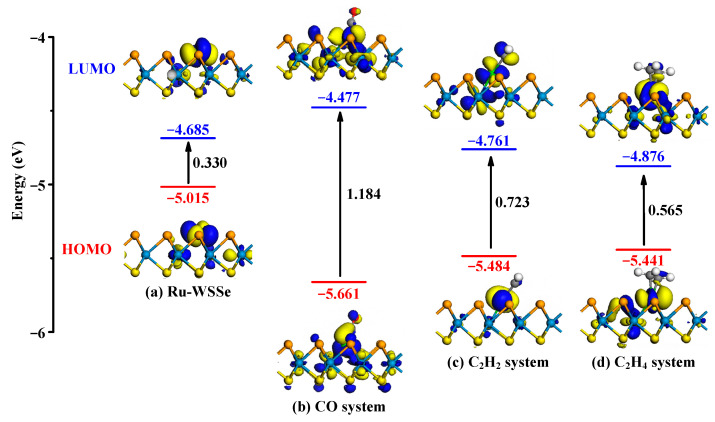
Distributions and energy levels of the HOMO and LUMO for both the isolated and gas adsorbed Ru-WSSe monolayers.

In this work, we identify $\sigma_{\mathrm{gas}}$ and $\sigma_{\mathrm{pure}}$ respectively as the electrical conductivity of Ru-WSSe/gas system and the isolated Ru-WSSe monolayer. Herein, we in Figure 7 present the spatial distributions and energy levels of the HOMO and LUMO for both the isolated and gas adsorbed Ru-WSSe monolayers. This visual representation is pivotal for understanding the variations in the energy gap that occur as a result of gas adsorption.

Figure 7a provides a detailed visualization of the HOMO and LUMO distributions in the Ru-WSSe monolayer, revealing that these orbitals are predominantly localized on the Ru atom. This observation suggests a high electron density around the Ru atom, marking it as a reactive center for gas adsorption. The calculated energies for the HOMO and LUMO in the Ru-WSSe system are −5.015 eV and −4.685 eV, respectively, yielding an initial energy gap of 0.330 eV. Upon exposure to CO, C_2_H_2_ and C_2_H_4_, the spatial distributions of the HOMO and LUMO are observed to undergo deformation, particularly around the Ru-C bonds as revealed in Figure 7b–d. This deformation is indicative of the strong orbital interactions that occur during gas adsorption. Consequently, the energies of the HOMO and LUMO in the gas adsorbed systems are altered, resulting in expanded energy gaps: 1.184 eV for CO system, 0.723 eV for C_2_H_2_ system, and 0.565 eV for C_2_H_4_ system. Thus, the energy gap of the Ru-WSSe monolayer is increased by 0.854 eV (258.8%) after adsorption of CO molecule, by 0.393 eV (119.9%) after adsorption of C_2_H_2_ molecule, and by 0.235 eV (71.2) after adsorption of C_2_H_4_ molecule. Based on these energy gap variations, the sensing responses of the Ru-WSSe monolayer to CO, C_2_H_2_, and C_2_H_4_ molecules are calculated to be 1.67 × 10^6^, 2.10 × 10^5^, and 9.61 × 10^3^, respectively. These substantial sensing responses indicate that the electrical resistance of the isolated Ru-WSSe monolayer is relatively low, and the adsorption of these gas species significantly increases its electrical resistance. This increase in resistance leads to a heightened sensing response, facilitating easy detection in practical applications [55]. 

### 3.5. Gas Sensing and Operation Mechanisms

Upon analysis of the Ru-WSSe monolayer’s exceptional sensing capabilities towards typical dissolved gases in transformer oil, it becomes imperative to delve deeper into the gas sensing mechanism. This exploration should be aligned with the operational principles to bolster its potential for real-world applications. Our initial step involves calculating the BS of the unmodified WSSe monolayer, which reveals a bandgap of 1.740 eV. Notably, the Fermi level is observed to be significantly closer to the valence band, underscoring the p-type semiconducting nature of the WSSe monolayer in this study. This observation is in excellent agreement with the bandgap of 1.71 eV reported in Reference [38]. Furthermore, the Fermi level in the Ru-WSSe monolayer is found to be even closer to the valence band, suggesting that the introduction of Ru leads to a substantial p-type doping effect on the pristine surface. This doping results in the Ru-WSSe monolayer also behaving p-type semiconducting properties. In gas adsorptions, the three targeted gas species—CO, C_2_H_2_, and C_2_H_4_—extract electrons from the Ru-WSSe monolayer. This electron-donating behavior of the monolayer leads to a reduction in carrier density, consequently increasing the electrical resistance in the presence of these gas species, consistent with the enhanced bandgap in the gas adsorbed systems. It is also crucial to highlight that the electron-withdrawing nature of C_2_H_2_ and C_2_H_4_ has been corroborated in Ref. [56], where Pt-doped AlNNTs acts as electron donors. Conversely, it should be noted that these three gas species can exhibit electron-donating properties under certain conditions. For instance, our previous research has demonstrated that CO, C_2_H_2_, and C_2_H_4_ can release electrons to nano surfaces including the Pd-doped MoSe_2_ [16] and Ni-doped WTe_2_ [57]. Therefore, one can assume that the electron-transfer behavior between the nano surface and the gas species is attributed to their electronic interactions, wherein the doped atoms may play the dominant role. Since metal-doping can tune the electronic property of the surface such as the work function, then a surface with a higher work function is more likely to accept electrons, while a surface with a lower work function may facilitate electron donation [58]. Therefore, different adsorption systems can have a completely opposite charge transfer path between the surface and the gas species. 

**Figure 8:**
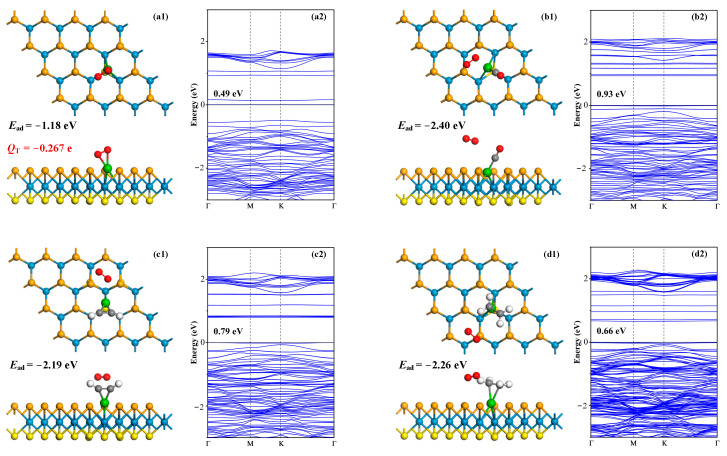
Adsorption of O_2_, co-adsorption of O_2_ and typical gases on Ru-WSSe monolayer and related BS. In BS, the black values are bandgap. (**a1**,**a2**) O_2_ system, (**b1**,**b2**) O_2_@CO system, (**c1**,**c2**) O_2_@C_2_H_2_ system and (**d1**,**d2**) O_2_@C_2_H_4_ system.

Given the widespread practice of DGA for electrical transformers, which involves extracting dissolved gases from the oil and subsequently detecting their presence, the influence of atmospheric O_2_ in the gas detection cannot be disregarded. To address this, we have conducted simulations of O_2_ molecule adsorption, as well as the co-adsorption of O_2_ with the three aforementioned gas molecules onto the Ru-WSSe monolayer. This approach aims to elucidate the impact of O_2_ on the adsorption and sensing performance of the proposed monolayer, thereby enhancing our understanding of its potential in practical sensing applications. The adsorption configurations and related BS are depicted in Figure 8.

For single O_2_ molecule adsorption onto the Ru-WSSe monolayer, it is evident that the O_2_ molecule forms two Ru-O bonds with the Ru dopant, signifying that the O_2_ molecule is effectively captured by the Ru atom. The *E*_ad_ in this systems is calculated as −1.18 eV, confirming the good binding affinity of the Ru-WSSe monolayer upon the O_2_ molecule, although this is not stronger than the binding with the three dissolved gas species. Besides, the adsorbed O_2_ molecule exhibits an electron-donating property, resulting in a negative charge of −0.267 e. Consequently, the bandgap of the O_2_ adsorbed system is determined to be 0.49 eV, which is a slight expansion compared to the 0.36 eV of the isolated Ru-WSSe monolayer. This augmentation in bandgap upon O_2_ adsorption can be ascribed to the p-type semiconductor attribute of the monolayer, which accepts electrons from the gas species, thereby escalating its electrical resistance.

In the scenario of co-adsorption of O_2_ with the three typical gas species, it is observed that O_2_ molecule keeps a relatively longer distance from the Ru dopant, revealing that the three typical gases exhibit the stronger binding affinity with the Ru-WSSe monolayer relative to the O_2_ molecule. From the aspect of *E*_ad_, it can be inferred that the presence of the O_2_ molecule to some extent promotes the adsorption performance of the Ru-WSSe monolayer towards CO, C_2_H_2_, and C_2_H_4_. This enhancement is confirmed by the more negative values of *E*_ad_ obtained in the presence of O_2_, with calculations yielding −2.40 eV, −2.19 eV, and −2.26 eV for each respective gas. It is also crucial to acknowledge that the presence of the O_2_ molecule can exert an impact on the sensing performance of the Ru-WSSe monolayer. Generally, the bandgaps of the O_2_@CO, O_2_@C_2_H_2_, and O_2_@C_2_H_4_ co-adsorbed Ru-WSSe systems are calculated to be 0.93, 0.79, and 0.66 eV, respectively. This indicates that the bandgaps of the co-adsorbed systems are larger than that of the monolayer with single O_2_ adsorption, with the increased rate orderly as CO > C_2_H_2_ > C_2_H_4_. This order is equivalent with the changing rate in bandgap of gas adsorbed systems with the absence of O_2_ molecule. Accordingly, the sensing response of the Ru-WSSe monolayer in the presence of O_2_ molecule may be somewhat diminished, with responses calculated to be 5.25 × 10^5^, 3.34 × 10^4^, and 2.64 × 10^3^ for CO, C_2_H_2_, and C_2_H_4_, respectively. These values are derived from Eequations (3) and (4), where the energy gap is represented here by the bandgap. Nonetheless, such sensing responses are still significant for the detection of these three gases, which are substantial and readily detectable using an electrochemical workstation [59]. These analyses in realm of the gas adsorption and sensing performances of the Ru-WSSe monolayer in the presence of O_2_ provide valuable insights into the material’s potential for real gas sensing applications, which are crucial for various industrial and environmental monitoring applications. 

All in all, these findings underscore the potential of the Ru-WSSe monolayer as an exceptional resistive gas sensor for the detection of the three typical gas species extracted from the in oil-immersed transformers. Specifically, the electrical conductivity of the Ru-WSSe monolayer is found to decrease significantly in the presence of CO, C_2_H_2_, and C_2_H_4_. This property allows the material to be utilized for monitoring the operational status of oil-immersed transformers, providing a reliable and sensitive means of detecting partial discharge or partial overheat within the transformers. 

## 4. Conclusions

In this study, we harness first-principles computational insights to Ru-doping property within the pristine WSSe monolayer. We simulate the adsorption process and analyze the sensing capabilities of the Ru-WSSe monolayer when exposed to three typical fault gases—CO, C_2_H_2_, and C_2_H_4_—to demonstrate its potential as a gas sensor for the safety monitoring of oil-immersed transformers. Our key findings are as follows:

(I) The Ru dopant is found to preferentially substitute a Se atom within the WSSe monolayer, with an *E*_form_ of 0.01 eV, indicating a highly favorable and energetically stable doping process;
(II) The Ru-WSSe monolayer exhibits chemisorption towards the three gas species, with the *E*_ad_ following the order: CO (−2.22 eV) > C_2_H_2_ (−2.01 eV) > C_2_H_4_ (−1.70 eV);
(III) The adsorption of these gases significantly increases the energy gap of Ru-WSSe monolayer from its initial value of 0.330 eV to 1.184 eV for CO, 0.723 eV for C_2_H_2_, and 0.565 eV for C_2_H_4_, respectively, as elucidated by the FMO theory;
(IV) The impact of the O_2_ molecule on the gas adsorption and sensing performance of the Ru-WSSe monolayer with the three typical gas species is analyzed, revealing a weakened but still remarkable sensing response in the three gas systems.

These results highlight the robust potential of the Ru-WSSe monolayer as a resistive gas sensor for the dissolved gases in transformer oil. The substantial increase in the energy gap upon gas adsorption is expected to translate into a significant decrease in electrical conductivity, which is a desirable trait for sensitive and selective gas detection. We anticipate that this work will inspire further innovative and intriguing research on Janus TMDs for gas sensing applications, particularly within the power system industry.

## Data Availability

The data presented in this study are available on request from the corresponding author.
